# LncRNA HOXA11‐AS promotes hepatocellular carcinoma progression by repressing miR‐214‐3p

**DOI:** 10.1111/jcmm.13633

**Published:** 2018-05-15

**Authors:** Meixiao Zhan, Ke He, Jing Xiao, Fei Liu, Haihe Wang, Zhenglin Xia, Xiaopeng Duan, Rui Huang, Yong Li, Xu He, Hua Yin, Guoan Xiang, Ligong Lu

**Affiliations:** ^1^ Center of Intervention radiology Zhuhai Precision Medicine Center Zhuhai People's Hospital Zhuhai Guangdong China; ^2^ Department of General Surgery Guangdong Second Provincial General Hospital Southern Medical University Haizhu District, Guangzhou Guangdong Province China; ^3^ Department of Biochemistry Zhongshan School of Medicine Sun Yat‐sen University Guangzhou Guangdong China; ^4^ Department of Dental Medicine Nanfang Hospital of Southern Medical University Guangzhou Guangdong China

**Keywords:** hepatocellular carcinoma, HOXA11‐AS, LncRNA, miR‐214‐3p

## Abstract

Accumulating studies supported that lncRNAs played important roles in tumorigenesis. LncRNA HOXA11‐AS was a novel lncRNA that has been proved to involved in several tumours. However, the role of HOXA11‐AS in the development of hepatocellular carcinoma (HCC) remains to be explained. In our study, we showed that HOXA11‐AS expression was up‐regulated in the HCC tissues, and the higher expression of HOXA11‐AS was associated with the advanced stage in the HCC samples. In addition, we indicated that the expression of HOXA11‐AS was up‐regulated in HCC cell lines (Hep3B, SMMC‐7721, MHCC97‐H and BEL‐7402) compared with normal liver cell lines (HL‐7702). Overexpression of HOXA11‐AS promoted HCC proliferation and invasion and induced the epithelial‐mesenchymal transition (EMT) and knockdown of HOXA11‐AS suppressed the HCC cell proliferation and invasion. However, we showed that miR‐214‐3p expression was down‐regulated in the HCC tissues and cell lines. Ectopic expression of miR‐214‐3p suppressed HCC cell proliferation and invasion. Furthermore, we indicated that overexpression of HOXA11‐AS decreased the miR‐214‐3p expression and the expression of miR‐214‐3p was negatively related with the HOXA11‐AS expression in HCC samples. Ectopic expression of HOXA11‐AS increased HCC proliferation and invasion and induced EMT through inhibiting miR‐214‐3p expression. These data suggested that HOXA11‐AS/miR‐214‐3p axis was responsible for development of HCC.

## 1 INTRODUCTION

Hepatocellular carcinoma (HCC) is one of most common tumour worldwide and is the 2nd leading cause of cancer‐related death.^1^, ^2^, ^3^, ^4^, ^5^ The high mortality rate of this disease is due to lack of impactful treatments.^6^, ^7^, ^8^ Despite interventional therapy, liver transplantation, chemotherapy and surgery could cure HCC cases successfully; the 5‐year survival rate was still dissatisfied.^9^, ^10^, ^11^ Hepatocarcinogenesis was one complicated procedure and that several factors were involved in the initiation, development and progression of this disease.^12^, ^13^, ^14^ However, the detail molecular mechanism of HCC remains largely unknown.^15^ Therefore, it is crucial to unravel the molecular mechanism and find the diagnostic markers for HCC.

Long non‐coding RNAs (lncRNAs) are one type of non‐coding RNA that modulates gene expression at post‐transcriptional or transcriptional level with the length more than 200 nucleotides.^16^, ^17^, ^18^ Emerging studies indicated that lncRNA acts critical roles in a wide range of cell processes such as cell cycle, differentiation, proliferation, migration, metabolism and apoptosis.^19^, ^20^ LncRNAs can serve as oncogenes or tumour suppressor genes via crosstalk with other RNA or by several chromatin‐based mechanisms.^21^, ^22^, ^23^ The expression of lncRNAs was found to be deregulated in a large number of tumours including lung cancer, osteosarcoma, ovarian cancer, breast cancer, gastric cancer, colorectal cancer and HCC.^24^, ^25^, ^26^, ^27^, ^28^, ^29^, ^30^ LncRNA HOXA11‐AS was a novel lncRNA that has been proved to involved in development of some tumours such as gastric cancer, lung cancer and osteosarcoma.^31^, ^32^, ^33^ However, the role of lncRNA HOXA11‐AS in the HCC remains largely unknown.

In this study, we investigated the expression and the functional role of HOXA11‐AS in HCC. We demonstrated that the expression of HOXA11‐AS was down‐regulated in HCC cell lines and tissues and overexpression of HOXA11‐AS promoted HCC growth, invasion and epithelial‐mesenchymal transition (EMT).

## 2 MATERIALS AND METHODS

### 2.1 Samples

A total of 40 pairs of HCC samples and the adjacent noncancerous samples were selected from cases undergone surgery at the Guangdong Second Provincial General Hospital. All tissues were obtained with written informed consent of each patient, and our study was approved by Review Board of Guangdong Second Provincial General Hospital. Tissues were snapping frozen in the liquid nitrogen until protein or RNA extraction. Both non‐tumour and tumour samples were histologically confirmed.

### 2.2 Cell lines and culture and transfection

Four human HCC cell lines (MHCC‐97H, Bel‐7404, SMMC7721 and QGY‐7703) and one normal liver cell line (HL‐7702) were purchased from the Cell Biology of Chinese Academy of Science from Shanghai. These cells were maintained in the 1640 medium (Invitrogen, CA, USA) supplemented with FBS and penicillin/treptomycin. pcDNA‐HOXA11‐AS and pcDNA‐control, siRNA‐control, siRNA‐HOXA11‐AS, miR‐214‐3p miminc and scramble were purchased from RiboBio (Guangzhou, China) and transfected to the cells using Lipofectamine 2000 kit (Invitrogen) according to the protocol.

### 2.3 RNA extraction and quantitative RT‐PCR

Total sample and cell RNA were extracted with TRIzol kit (Invitrogen), and then cDNA was synthesized following to manufacturer's information. Quantitative real‐time PCR (qRT‐PCR) was conducted to determine the mRNA and lncRNA using SYBR Green Master Mix (MBI Fermentas) on the IQ5 PCR machine. The relative expression level of gene was calculated by the 2^−(DDCt)^ method. The primers in our manuscript were used as following: HOXA11‐AS‐F 5′‐GAGTGTTGGCCTGTCCTCAA‐3′, HOXA11‐AS‐R 5′‐TTGTGCCCAGTTGCCTGTAT‐3′; miR‐214‐3p‐F 5′‐GAGTGTTGGCCTGTCCTCAA‐3′, miR‐214‐3p‐R 5′‐TTGTGCCCAGTTGCCTGTAT‐3′. The mRNA and miRNA expression levels were normalized to GAPDH and RNU6B expression levels, respectively.

### 2.4 Cell proliferation assay and invasion assay

3‑(4,5‑dimethylthiazol‑2‑yl)‑2,5‑diphenyltetrazolium (MTT; Sigma) was used to determine the cell proliferation. Cells were cultured in the 96‑well plate for 0, 24, 48 and 72 hours. The absorbance at the 540 nm was determined at the spectrophotometer. Cell invasion was conducted using Transwell chamber coated with Matrigel (BD Biosciences, USA). Cells were cultured on the upper chamber in serum‐free 1640 medium. As chemo‐attractant, 1640 medium containing 20% FBS was put to the lower chamber. Invaded cell was stained with crystal violet and photographed (Olympus, Japan).

### 2.5 Western blot analysis

Protein from HCC cells or samples was extracted using RIPA buffer with the proteinase inhibitor. The concentration of protein was evaluated by the Bradford kit (Bio‐Rad, CA). Twenty micrograms protein lysate was resolved by 10% SDS‐PAGE and electrophoretically transferred to polyvinylidene difluoride (PVDF). The membrane was blotted with the primary antibodies (anti‐MELK, anti‐E‐cadherin, N‐cadherin, vimentin and Snail antibodies, Abcam). The secondary antibody was IgG‐HRP, and immunoreactive band was visualized by enhanced chemiluminescence detection system.

### 2.6 Statistical analysis

The data were expressed as the mean ± standard deviation (SD). Statistical analysis was conducted by SPSS 17.0 (IBM, NY, USA). The Student's *t* test was performed to assess significance of differences between 2 groups. ANOVA was used assess significance of differences between more than 2 groups. *P* < .05 was determined as significant difference.

## 3 RESULT

### 3.1 HOXA11‐AS was up‐regulated in HCC samples

**Figure 1:**
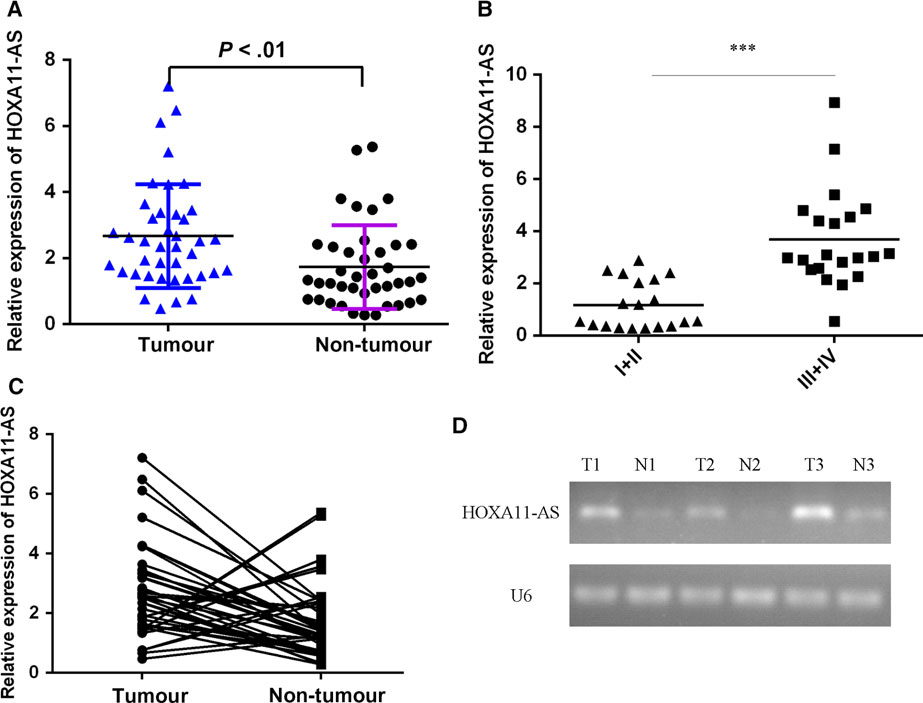
HOXA11‐AS was up‐regulated in HCC samples. A, The expression of HOXA11‐AS in tumorous samples and compared normal samples of 40 patients was determined by qRT‐PCR. U6 was used as the internal control. B, The expression of HOXA11‐AS was lower in initial clinical stage (I‐II phase) cases than that advanced clinical stage (III‐IV phase) cases. C, Of 40 HCC samples, HOXA11‐AS was up‐regulated in 20 patients (20/30, 67%) compared with adjacent normal tissues. D, The expression of HOXA11‐AS in tumorous samples and compared normal samples of 3 HCC patients was detected by RT‐PCR. ***P < .001

The expression of HOXA11‐AS was examined using qRT‐PCR in tumorous samples and compared normal samples of 40 patients. As shown in the Figure 1A, HOXA11‐AS level was higher in tumorous samples than that of compared normal tissues. In addition, expression of HOXA11‐AS was lower in initial clinical stage (I‐II phase) cases than that advanced clinical stage (III‐IV phase) cases (Figure 1B). Of 40 HCC samples, HOXA11‐AS was up‐regulated in 20 patients (20/30, 67%) compared with adjacent normal tissues (Figure 1C). Moreover, we found that the expression of HOXA11‐AS was up‐regulated in the HCC tissues compared with in normal tissues using RT‐PCR (Figure 1D).

### 3.2 miR‐214‐3p was down‐regulated in HCC samples

**Figure 2:**
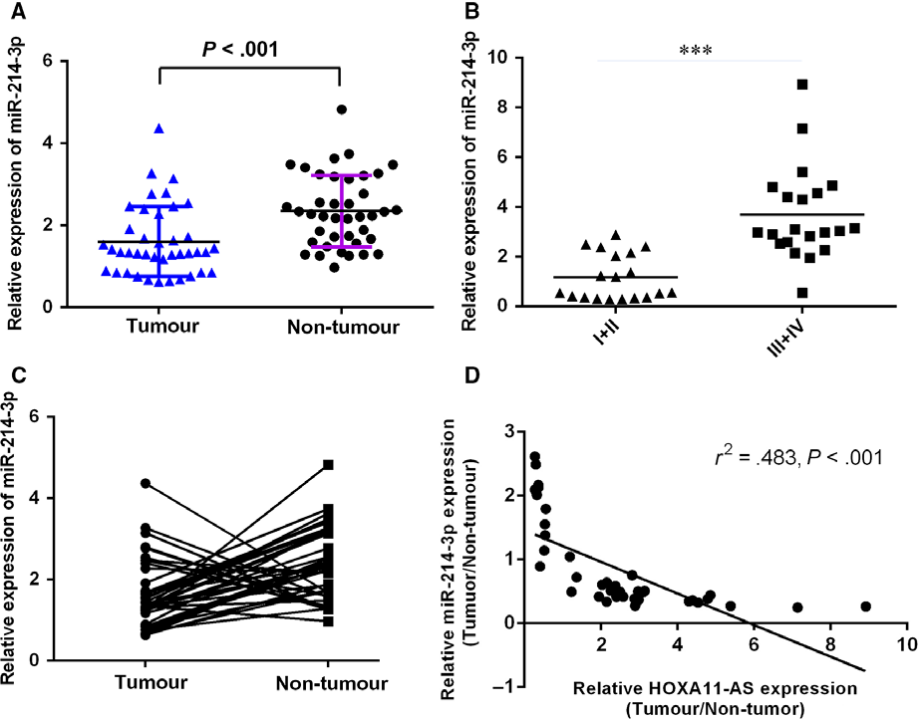
miR‐214‐3p was down‐regulated in HCC samples. A, The expression of miR‐214‐3p in tumorous samples and compared normal samples of 40 patients was determined by qRT‐PCR. U6 was used as the internal control. B, The expression of miR‐214‐3p was higher in initial clinical stage (I‐II phase) cases than that advanced clinical stage (III‐IV phase) cases. C, Of 40 HCC samples, miR‐214‐3p was down‐regulated in 20 patients (20/30, 67%) compared with adjacent normal tissues. D, The expression of miR‐214‐3p was negatively related with the HOXA11‐AS expression in HCC samples. ***P < .001

The expression of miR‐214‐3p was measured using qRT‐PCR in tumorous samples and compared normal samples of 40 patients. As shown in the Figure 2A, miR‐214‐3p level was lower in tumorous samples than that of compared normal tissues. In addition, expression of miR‐214‐3p was higher in initial clinical stage (I‐II phase) cases than that advanced clinical stage (III‐IV phase) cases (Figure 2B). Of 40 HCC samples, miR‐214‐3p was down‐regulated in 20 patients (20/30, 67%) compared with adjacent normal tissues (Figure 2C). Moreover, we indicated that the expression of miR‐214‐3p was negatively related with the HOXA11‐AS expression in HCC samples (Figure 1D).

### 3.3 Overexpression of HOXA11‐AS promoted HCC proliferation and invasion

**Figure 3:**
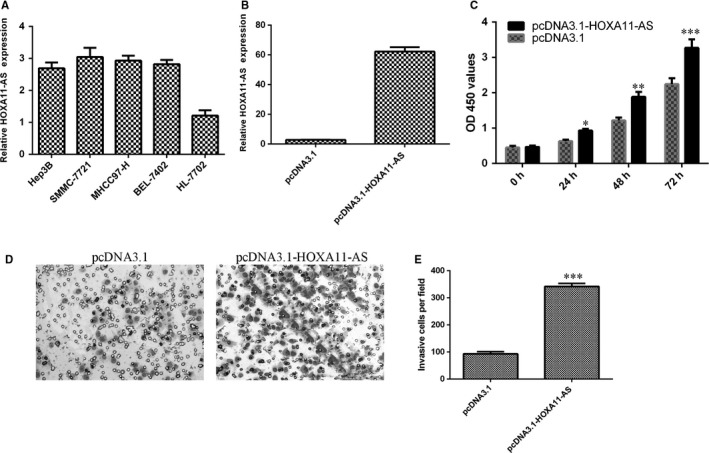
Overexpression of HOXA11‐AS promoted HCC proliferation and invasion. A, The expression of HOXA11‐AS in the HCC cell lines (Hep3B, SMMC‐7721, MHCC97‐H and BEL‐7402) and normal liver cell lines (HL‐7702) was measured by qRT‐PCR. B, HOXA11‐AS expression in the SMMC‐7721 cell was detected by qRT‐PCR. C, Ectopic expression of HOXA11‐AS promoted the SMMC‐7721 cell proliferation. D, Overexpression of HOXA11‐AS increased the cell invasion in the SMMC‐7721 cell. E, The relative invasive cell numbers were shown. *P < .05, **P < .01 and ***P < .001

In the HCC cell lines (Hep3B, SMMC‐7721, MHCC97‐H and BEL‐7402), HOXA11‐AS expression was up‐regulated compared with normal liver cell lines (HL‐7702) (Figure 3A). Then, in SMMC‐7721 transfected with pcDNA3.1‐HOXA11‐AS, HOXA11‐AS expression was increased compared with control group (Figure 2B). Ectopic expression of HOXA11‐AS promoted the SMMC‐7721 cell proliferation (Figure 3C). Overexpression of HOXA11‐AS increased the cell invasion in the SMMC‐7721 cell (Figure 3D), and the relative invasive cells are shown in Figure 3E.

### 3.4 Knockdown of HOXA11‐AS suppressed HCC proliferation and invasion

**Figure 4:**
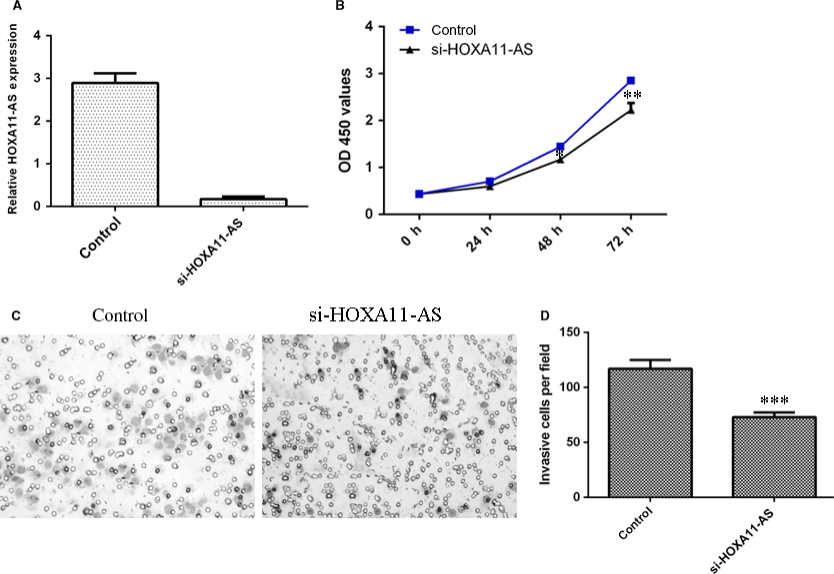
Knockdown of HOXA11‐AS suppressed HCC proliferation and invasion. A, HOXA11‐AS expression in the SMMC‐7721 cell was detected by qRT‐PCR. B, Knockdown of HOXA11‐AS suppressed the SMMC‐7721 cell growth. C, Inhibition of HOXA11‐AS decreased the cell invasion in the SMMC‐7721 cell. D, The relative invasive cells were shown. *P < .05, **P < .01 and ***P < .001

Then, in SMMC‐7721 transfected with siRNA‐HOXA11‐AS, HOXA11‐AS expression was down‐regulated compared with control group (Figure 4A). Knockdown of HOXA11‐AS suppressed the SMMC‐7721 cell growth (Figure 4B). Inhibition of HOXA11‐AS decreased the cell invasion in the SMMC‐7721 cell (Figure 4C), and the relative invasive cells are shown in Figure 4D.

### 3.5 Ectopic expression of HOXA11‐AS induced epithelial‐mesenchymal transition (EMT)

**Figure 5:**
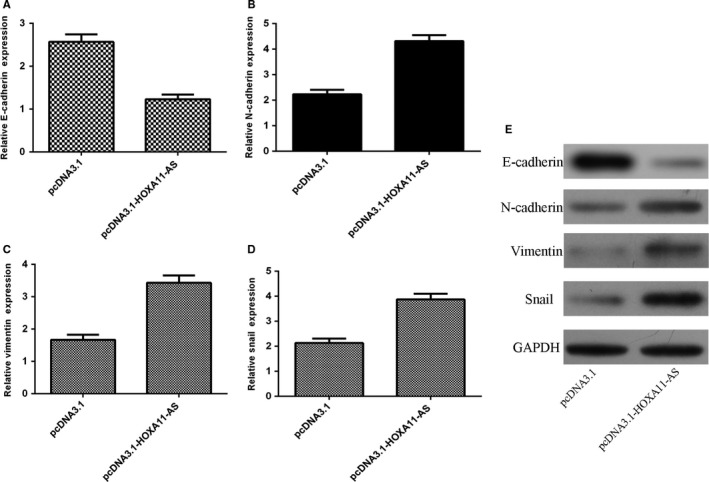
Ectopic expression of HOXA11‐AS induced epithelial‐mesenchymal transition (EMT). A, The mRNA expression of E‐cadherin was determined by qRT‐PCR. B, Ectopic expression of HOXA11‐AS increased the N‐cadherin expression in the SMMC‐7721 cell. C, The mRNA expression of vimentin was measured by qRT‐PCR. D, Overexpression of HOXA11‐AS induced the Snail expression in the SMMC‐7721 cell. E, The protein expression of E‐cadherin, N‐cadherin, vimentin and Snail was determined by Western blot. GAPDH was used as the control

Overexpression of HOXA11‐AS suppressed E‐cadherin expression in the SMMC‐7721 cell (Figure 5A). Ectopic expression of HOXA11‐AS increased the N‐cadherin expression in the SMMC‐7721 cell (Figure 5B). HOXA11‐AS overexpression promoted the vimentin expression in the SMMC‐7721 cell (Figure 5C). Ectopic expression of HOXA11‐AS induced the Snail expression in the SMMC‐7721 cell (Figure 5D). In addition, we confirmed that ectopic expression of HOXA11‐AS decreased the protein expression of E‐cadherin and increased the N‐cadherin, vimentin and Snail expression in the SMMC‐7721 cell (Figure 5E).

### 3.6 Ectopic expression of miR‐214‐3p suppressed HCC proliferation and invasion

**Figure 6:**
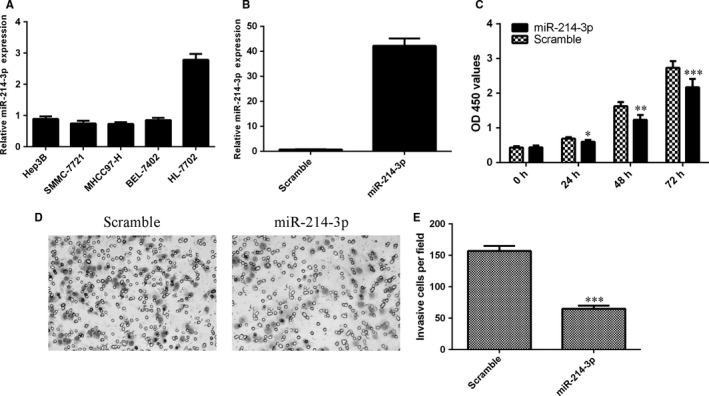
Ectopic expression of miR‐214‐3p suppressed HCC proliferation and invasion. A, The expression of miR‐214‐3p in the HCC cell lines (Hep3B, SMMC‐7721, MHCC97‐H and BEL‐7402) and normal liver cell lines (HL‐7702) was measured by qRT‐PCR. B, The expression of miR‐124‐3p in the SMMC‐7721 cell was measured by qRT‐PCR. C, Ectopic expression of miR‐124‐3p suppressed the SMMC‐7721 cell proliferation. D, The relative invasive cell numbers were shown. *P < .05, **P < .01 and ***P < .001

In the HCC cell lines (Hep3B, SMMC‐7721, MHCC97‐H and BEL‐7402), miR‐214‐3p expression was down‐regulated compared with normal liver cell lines (HL‐7702) (Figure 6A). Then, in SMMC‐7721 transfected with miR‐124‐3p mimics, miR‐124‐3p expression was increased compared with control group (Figure 6B). Ectopic expression of miR‐124‐3p decreased the SMMC‐7721 cell proliferation (Figure 6C). Overexpression of miR‐124‐3p suppressed the cell invasion in the SMMC‐7721 cell (Figure 6D), and the relative invasive cells are shown in Figure 6E.

### 3.7 HOXA11‐AS overexpression suppressed the miR‐214‐3p expression in HCC cell

**Figure 7:**
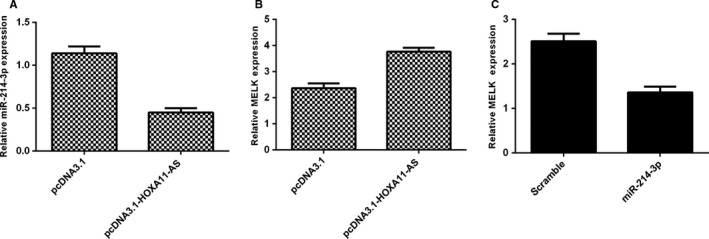
HOXA11‐AS overexpression suppressed the miR‐214‐3p expression in HCC cell. A, The miR‐214‐3p expression was measured by qRT‐PCR in the SMMC‐7721 cell. B, HOXA11‐AS overexpression enhanced the MELK expression in the SMMC‐7721 cell. C, Overexpression of miR‐214‐3p decreased the MELK expression in the SMMC‐7721 cell

Ectopic expression of HOXA11‐AS inhibited the miR‐214‐3p expression in SMMC‐7721 cell (Figure 7A). HOXA11‐AS overexpression enhanced the MELK expression in the SMMC‐7721 cell (Figure 7B). Overexpression of miR‐214‐3p decreased the MELK expression in the SMMC‐7721 cell (Figure 7C).

### 3.8 HOXA11‐AS overexpression promoted HCC proliferation and invasion and induced EMT through inhibiting miR‐214‐3p expression

**Figure 8:**
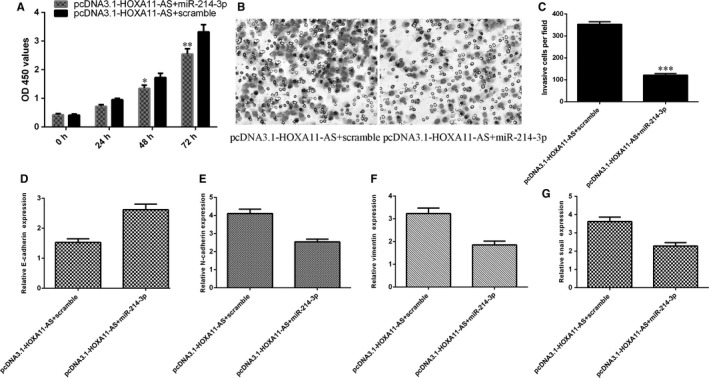
HOXA11‐AS overexpression promoted HCC proliferation and invasion and induced EMT through inhibiting miR‐214‐3p expression. A, Ectopic expression of miR‐214‐3p attenuated the proliferation effect of HOXA11‐AS in the SMMC‐7721 cells. B, Overexpression of miR‐214‐3p decreased the invasion ability of HOXA11‐AS overexpressing SMMC‐7721 cells. C, The relative invasive cell numbers were shown. D, The expression of E‐cadherin was detected by qRT‐PCR. E, Ectopic expression of miR‐214‐3p decreased the N‐cadherin expression in the HOXA11‐AS overexpressing SMMC‐7721 cell. F, The mRNA expression of vimentin was detected by qRT‐PCR. G, The mRNA expression of Snail was detected by qRT‐PCR. *P < .05, **P < .01 and ***P < .001

To determine the role of miR‐214‐3p in the HOXA11‐AS‐promoting HCC progression, we transfected miR‐214‐3p mimic in HOXA11‐AS overexpressing SMMC‐7721 cells. We found that ectopic expression of miR‐214‐3p attenuated the proliferation effect of HOXA11‐AS in the SMMC‐7721 cells (Figure 8A). Furthermore, we indicated that overexpression of miR‐214‐3p decreased the invasion ability of HOXA11‐AS overexpressing SMMC‐7721 cells (Figure 8B) and the relative invasive cells were shown (Figure 8C). Overexpression of miR‐214‐3p enhanced the E‐cadherin expression in the HOXA11‐AS overexpressing SMMC‐7721 cell (Figure 8D). Ectopic expression of miR‐214‐3p decreased the N‐cadherin expression in the HOXA11‐AS overexpressing SMMC‐7721 cell (Figure 8E). Overexpression of miR‐214‐3p suppressed the vimentin (Figure 8F) and Snail (Figure 8G) in the HOXA11‐AS overexpressing SMMC‐7721 cell.

## 4 DISCUSSION

In this study, we demonstrated that the expression of HOXA11‐AS was up‐regulated in the HCC samples and the higher expression of HOXA11‐AS was associated with the advanced stage in the HCC samples. However, we showed that miR‐214‐3p expression was down‐regulated in the HCC tissues and the lower expression of miR‐214‐3p was correlated with advanced stage in the HCC tissues. Overexpression of HOXA11‐AS promoted HCC proliferation and invasion and induced the epithelial‐mesenchymal transition (EMT). HOXA11‐AS overexpression suppressed the miR‐214‐3p expression and enhanced the MELK expression. HOXA11‐AS overexpression promoted HCC proliferation and invasion and induced EMT through inhibiting miR‐214‐3p expression. These data suggested that HOXA11‐AS/miR‐214‐3p axis was responsible for development of HCC.

Previous evidences have proved that lncRNA HOXA11‐AS expression was found to be overexpressed and played an oncogene in several tumours.^34^, ^35^, ^36^, ^37^, ^38^ Xu et al^39^ showed that HOXA11‐AS expression was up‐regulated in glioma cell lines and samples. Knockdown expression of HOXA11‐AS suppressed glioma cell invasion, migration and growth partly through promoting miR‐214‐3p expression. Chen et al^34^ demonstrated that HOXA11‐AS increased EMT through regulating miR‐200b expression in the non‐small‐cell lung cancer (NSCLL). Wang et al^40^ indicated that ectopic expression of HOXA11‐AS induced the glioma cell growth and knockdown of HOXA11‐AS decreased the cell growth. Liu et al^32^ showed that HOXA11‐AS expression was up‐regulated in gastric cancer tissues and HOXA11‐AS knockdown decreased gastric cancer cell cycle and inhibited gastric cancer cell invasion, metastasis and migration through regulating β‐catenin and KLF2. In this study, the HOXA11‐AS expression in 40 paired primary HCC tissues and HCC cell lines was up‐regulated compared with adjacent normal samples and normal liver cell lines (HL‐7702). Moreover, ectopic expression of HOXA11‐AS promoted the HCC cell growth, invasion and EMT and knockdown of HOXA11‐AS suppressed the HCC cell proliferation and invasion. These data suggested that HOXA11‐AS might act as oncogene in HCC.

The underlying mechanism by how HOXA11‐AS involved in HCC tumorigenesis still remains to be researched. Growing studies indicated that lncRNAs acted crucial roles in several biological processes by playing as ceRNAs (competing endogenous RNAs) or molecular sponges to modulate‐specific miRNAs.^34^, ^37^, ^41^, ^42^, ^43^ Previous study showed that HOXA11‐AS functions as an oncogenic gene which enhanced glioma cell metastasis and growth by inhibiting miR‐214‐3p/EZH2 expression.^39^ In this regard, we also found that ectopic expression of HOXA11‐AS suppressing the miR‐214‐3p expression in the HCC cell. miR‐214 was demonstrated to be one tumour suppressor miRNA in a lot of tumours including HCC.^44^, ^45^, ^46^, ^47^ Yang et al^48^ indicated that miR‐214 inhibited the HCC cell proliferation through targeting E2F3 expression. Wang et al^49^ also confirmed that the expression of miR‐214 was down‐regulated in HCC specimens and cells and overexpression of miR‐214 suppressed HCC cell proliferation by regulating β‐catenin expression. In addition, Li et al^50^ showed that the expression of miR‐214‐3p was down‐regulated in HCC tissues and miR‐214‐3p overexpression decreased HCC cell cycle, cell proliferation and induced cell apoptosis partly by regulating embryonic leucine zipper kinase (MELK) expression. Therefore, we focused on the miR‐214‐3p as a ceRNA of HOXA11‐AS. We demonstrated that miR‐214‐3p expression was down‐regulated in the HCC tissues and the expression of miR‐214‐3p was negatively correlated with the expression of HOXA11‐AS. HOXA11‐AS overexpression promoted HCC proliferation and invasion and induced EMT through inhibiting miR‐214‐3p expression. These results suggested that lncRNA HOXA11‐AS played an oncogene role in HCC through acting as ceRNA for miR‐214‐3p.

From this project, we indicated that the expression of HOXA11‐AS was up‐regulated in the HCC samples and cell lines. Overexpression of HOXA11‐AS promoted HCC cell proliferation, invasion and EMT partly through inhibiting miR‐214‐3p expression. These data suggested that HOXA11‐AS may be a potential target for HCC treatment.

## CONFLICT OF INTEREST STATEMENT

There is no conflict of interest statement.
